# Adenovirus Specific Pre-Immunity Induced by Natural Route of Infection Does Not Impair Transduction by Adenoviral Vaccine Vectors in Mice

**DOI:** 10.1371/journal.pone.0145260

**Published:** 2015-12-17

**Authors:** Bruna de Andrade Pereira, Leoneide E. Maduro Bouillet, Natalia A. Dorigo, Cornel Fraefel, Oscar Bruna-Romero

**Affiliations:** 1 Department of Microbiology, Federal University of Minas Gerais, Belo Horizonte, MG, Brazil; 2 Institute of Virology, University of Zürich, Zürich, Switzerland; Boston College, UNITED STATES

## Abstract

Recombinant human adenovirus serotype 5 (HAd5V) vectors are gold standards of T-cell immunogenicity as they efficiently induce also humoral responses to exogenous antigens, in particular when used in prime-boost protocols. Some investigators have shown that pre-existing immunity to adenoviruses interferes with transduction by adenoviral vectors, but the actual extent of this interference is not known since it has been mostly studied in mice using unnatural routes of infection and virus doses. Here we studied the effects of HAd5V-specific immune responses induced by intranasal infection on the transduction efficiency of recombinant adenovirus vectors. Of interest, when HAd5V immunity was induced in mice by the natural respiratory route, the pre-existing immunity against HAd5V did not significantly interfere with the B and T-cell immune responses against the transgene products induced after a prime/boost inoculation protocol with a recombinant HAd5V-vector, as measured by ELISA and in vivo cytotoxic T-cell assays, respectively. We also correlated the levels of HAd5V-specific neutralizing antibodies (Ad5NAbs) induced in mice with the levels of Ad5NAb titers found in humans. The data indicate that approximately 60% of the human serum samples tested displayed Ad5NAb levels that could be overcome with a prime-boost vaccination protocol. These results suggest that recombinant HAd5V vectors are potentially useful for prime-boost vaccination strategies, at least when pre-existing immunity against HAd5V is at low or medium levels.

## Introduction

Vectors based on replication-defective recombinant human serotype 5 adenoviruses (HAd5V) are currently being used as experimental vaccines in pre-clinical and clinical studies, where they have repeatedly induced potent immune responses to transgene products. Studies performed by our group using HAd5V vectors for vaccination against parasitic infectious diseases such as Malaria, Toxoplasmosis, Chagas’ disease or Leishmaniosis[1–5] demonstrated that adenoviruses remain one of the most efficient vectors to induce T cell responses, even after a single inoculation, when compared to DNA vaccines, other viral vectors and synthetic peptide vaccines. [6]

HAd5-specific neutralizing antibodies (NAbs) are present in various levels in humans. Currently available data indicate a prevalence of Ad5NAbs ranging from 45% in populations of developed countries in North America and Europe to over 80% in some developing countries in Africa.[7–13] Some researchers have suggested that such prevalence of pre-existing immunity may blunt any attempt of broad use of HAd5V vectors as vaccines, as the vector would be eliminated before delivering the transgene for antigen synthesis and presentation. Despite this concern, experimental use of HAd5V vectors is still ongoing for two main reasons: (i) Successful immunizations and protection in spite of the presence of Ad5NAbs models have been reported in animals[14–17] as well as in humans. [18] (ii) Despite the lack of protection and higher seroconversion rates observed in participants of an HIV vaccine phase IIb trial conducted by Merck (STEP study) that used a recombinant HAd5V vector known as MRKAd5 to express HIV-1 gag/pol/nef, the results of that trial showed that an overall 77% of individuals were successfully immunized after inoculation of not one but three doses of the vaccine. [19] It is important to note that 86% of the individuals with none (< 20 neutralizing unit—NU) or low (< 200 NU) anti-adenoviral NAb titers and 68% of individuals with high (> 200 NU) anti-adenoviral NAb titers could be immunized. The lack of efficacy of the MRKAd5 HIV-1 gag/pol/nef vaccine was possibly related to a narrow repertoire of HIV-specific CD8^+^ T cell responses induced to recognize epitopes within the transmitting viral strains rather than associated with a lack of immunogenicity of the viral vector vaccine. In addition, subsequent studies showed no association between pre-existing HAd5V seropositivity (NAbs levels) and increased susceptibility to HIV infection in these individuals.[20] Furthermore, recent cancer gene therapy trials that used recombinant HAd5V vectors have repeatedly yielded positive results.[21,22,18,23] Altogether, prior data indicate that: (i) HAd5V vectors are efficient gene transfer vehicles in animal models as well as in humans and (ii) although adenoviral pre-existing immunity may interfere with transduction by adenoviral vectors, this interference may not be as pronounced as suggested in some animal models.

As a rule of thumb in Immunology, if a single dose of any antigen does not induce sufficient levels of immunogenicity and/or protection, a booster inoculation should be considered.[1,24] Following this, we previously described a very efficient heterologous HAd5V/poxvirus prime-boost immunization regime, yielding complete protection from infection by *Plasmodium yoelii*, the most infective of all rodent malaria parasites.[1] However, all two-dose homologous (HAd5V/HAd5V) prime/boost protocols tested were also very efficient in inducing secondary immune responses of higher magnitude against parasitic diseases,[1–4,25,26] thereby questioning the “consensus” theory of pre-existing immunity/vaccine-blockade.

Here we assessed the interference of specific Ad5NAbs induced by a “natural” infection on the efficiency of a homologous prime/boost immunization protocol. To this end, we reproduced in mice the anti-adenoviral humoral response generated by intranasal (natural infection route) or intramuscular (unnatural experimental route) inoculation. We demonstrate that a prime-boost immunization regime with HAd5V is able to induce efficient humoral and cellular immune responses against the transgene in mice with pre-existing anti-adenoviral humoral immunity generated by intranasal inoculation. By contrast, the pre-existing anti-adenoviral humoral responses generated by intramuscular inoculation did impair the immune response against the transgene regardless of the vaccination protocol used. Our results suggest that pre-existing anti-adenoviral humoral immunity does not preclude the use of recombinant adenoviral vaccine candidates in human trials.

## Materials and Methods

### Ethical Statement

Mice were purchased from the animal facilities (CEBIO) of the Federal University of Minas Gerais (Brazil) and used according to proceedings approved by the Ethical Committee for Experimental Animals of the University (CETEA-UFMG). The CETEA-UFMG approved this study. Serum samples were collected from the following sanitary institutions, Análises Clínicas (ANACLIN, Visconde do Rio Branco, Minas Gerais, Southeast of Brazil) and Celso Mattos laboratory (Santarém, Pará, North of Brazil). Sera were obtained and manipulated according to protocols approved by the Committees of Ethics in Research (COEPs) of the institutions involved. The protocols and procedures used to analyze the human sera were approved by the Ethical Committee of São João Batista Hospital (approval number 150307). Written informed consent from the donor was waived by the ethical committee once all human sera used in this study were primarily obtained for diagnostics in either of the institutions and donated for our study. In addition, human serum samples were analyzed anonymously. Ethical committee members: Dr. Ricardo Drei Valente, Dr. Reynaldo Sobral Neto, Dr. Otavio Coutinho de Almeida and Dr. Jose Slaibi.

### Animals, viruses and immunizations

Six-week-old female BALB/c mice were used in this study. Anti-HAd5V pre-immunity was induced by i.n. or i.m. routes with different doses (as indicated) of a GFP-recombinant HAd5V (Ad5GFP). Intranasal inoculation was performed in a final volume of 15 μl per nostril and i.m. was performed with 30 μl per quadriceps. Immunizations were performed under anesthesia. For experimental vaccination we used HAd5VCMV, a recombinant (ΔE1/E3) HAd5V previously constructed [20] that encodes a protective CD8+ T-cell epitope of the murine cytomegalovirus immediate early 1 (IE1 or pp89) antigen fused with the hepatitis B virus core protein (HBc). Immunizations with HAd5VCMV were performed with 1×10^9^ pfu/animal by s.c route. Ratios of plaque-forming units (pfu) to viral particles (vp) were always below 120 (range 35–120).

### *In vivo* cytotoxicity assay

Splenocytes of BALB/c mice depleted of erythrocytes were divided into two populations: (i) the first population (^low^CFSE, internal control) was labeled with a low concentration (6 μM) of fluorogenic dye carboxyfluorescein diacetate succinimidyl diester (CFSE) for 15 min at 37°C; (ii) the second population (^high^CFSE, target population) was labeled with a higher concentration (20 μM) of CFSE and further incubated with 4μg of synthetic peptide comprising the H-2L^d^-restricted epitope YPHFMPTNL for 30 min at 37°C. Equal amounts (7x10^6^ cells each) of both populations were i.v. injected in all experimental groups (100 μl/animal). The same cell suspension was used to inoculate all vaccinated and control animals. Twenty hours later, splenocytes from all groups were isolated and analyzed by flow cytometry. Specific lysis was calculated for each animal with the following formula: 100 –[(^high^CFSE × 100)/ ^low^CFSE]. Control groups (non immunized mice and mice immunized without pre-existing immunity) were considered as inducing 0% and 100% of specific lysis, respectively.

### Human sera

Two hundred and ten serum samples were collected from the following sanitary institutions, Análises Clínicas (ANACLIN, Visconde do Rio Branco, Minas Gerais, Southeast of Brazil – 103 samples) and Celso Mattos laboratory (Santarém, Pará, North of Brazil – 107 samples).

### Neutralizing antibody assay

HAd5V particles used for neutralization assays were purified in CsCl gradients or in adsorption filters (Vivapure adenopack 500, Sartorius, Germany) using standard protocols. Neutralizing antibody assays were performed as previously described.[26] In brief, serum samples were incubated with Ad5GFP at a multiplicity of infection of 6 for 1h at 37°C, and 100 μl of each sample were applied per 1×10^4^ 293 HEK (ATCC# CRL-1573) cells in 96-well plates (TPP, Switzerland). Serum samples were analyzed in 2-fold serial dilutions. The same controls (positive and negative sera) were included in all plates. The capacity of anti-AdH5V antibodies present in human sera to neutralize Ad5GFP particles was measured 14h after the initial virus/antibody contact by observation of GFP protein synthesis using fluorescence microscopy and, 48h later, by detection of cytopathic effects using light microscopy.

### Enzyme-linked immunosorbent assay (ELISA)

Sera of vaccinated mice were collected five days after the last immunization and analyzed for the presence of anti-HBc antibodies. Each serum was diluted (1:200) and added to a plate pre-coated with HBc antigen (Bioelisa anti-HBc kit, Spain). The mouse secondary antibody anti-IgG (Zymed, Life Technologies) was added to the wells and incubated for 1h at 37°C. After repetitive washes the reaction was developed using a chromogenic substrate (TMB) for 30 min at room temperature. Absorbance was measured at 450 nm in a Titertek microplate reader (McLean, VA).

### Statistical analysis

Data presented represents mean±standard deviation. Kruskal-Wallis analyses were performed to validate differences between experimental groups; p<0.05* was considered as significant, p<0.001** was considered as highly significant.

## Results

### Ability of adenovirus immunity to block transduction by adenoviral vectors

Our first aim was to determine in mice whether pre-existing immunity against adenovirus generated through intranasal (natural infection route) or intramuscular (unnatural experimental route) inoculation can impair the efficiency of an adenovirus vaccine vector. For this, mice were inoculated i.n. or i.m. with different doses of a control virus, Ad5GFP. These inoculation routes were chosen because most previous conclusions on the blocking capacity of pre-existing immunity were obtained from models in which parenteral routes were used,[27,9,28] despite the fact that humans get naturally infected with HAd5V by the respiratory route. Differences in the preferential type and intensity of immune responses induced by these two routes (mucosal vs. systemic) could account for the differences in vector efficiency observed in the previous studies. Four weeks after inoculation of different doses of Ad5GFP vectors (10^3^, 10^5^ and 10^7^ pfu), BALB/c mice were immunized with HAd5VCMV, a recombinant HAd5V that expresses a previously identified CD8+ T-cell immunogenic murine cytomegalovirus sequence included in the hepatitis B virus core protein (as a carrier virus-like particle).

**Fig 1:**
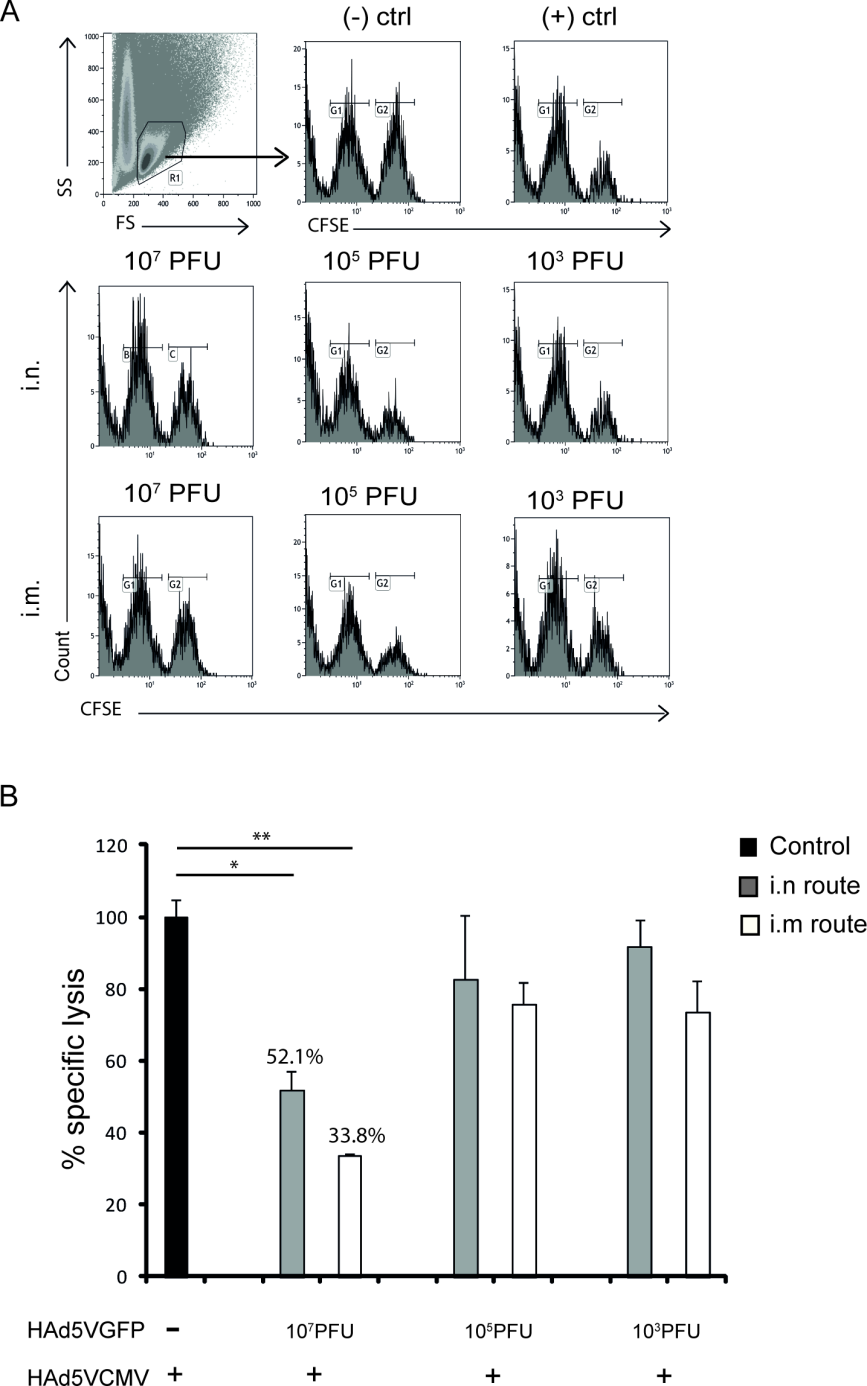
*In vivo* cytotoxicity levels against the murine CMV CD8 T-cell epitope YPHFMPTNL detected in animals with pre-existing HAd5V immunity or control animals after immunization with different doses of HAd5V-based vaccine vector, HAd5VCMV. Mice were immunized by either i.n. or i.m. routes with different doses of HAd5VGFP as indicated (10^3^, 10^5^ or 10^7^ pfu/animal). Four weeks later, mice were vaccinated with one (prime) dose of 1×10^9^ pfu/animal of HAd5VCMV administered subcutaneously. The ^low^CFSE and ^high^CFSE+^CMV^CD8-peptide labeled cells were identified by flow cytometry using the gates shown in panel (A). Histograms show data from a single mouse. FS–forward scatter, SS–side scatter. (B) Percentage of specific lysis induced in vaccinated animals after a single immunization (prime). The specific lysis was normalized to the positive control (set as 100% of specific lysis). Negative control represents mice that were not immunized with any adenovirus vector. The graph in panel (B) shows one of three independent experiments with at least 3 mice per group. Bars represent mean values +/- SD. (+) ctrl–positive control, (-) ctrl–negative control; (-) mice non-immunized with adenovirus; i.n.–intranasal immunization route; i.m.–intramuscular immunization route.

To analyze the effect of pre-existing immunity on adenoviral vaccination, we first used an ex *vivo* flow cytometry assay. This assay determined the antigen-specific cytotoxicity activity present in immunized animals by determining the amount of surviving CFSE-labelled antigen-incubated target cells versus control cells transferred to these mice 20h earlier. The gating strategy used to differentiate ^low^CFSE and ^high^CFSE+^CMV^CD8-peptide labeled cells is demonstrated in Fig 1A. Fig 1B confirms that, irrespective of the route used for pre-immunization, the immunogenicity of a single adenoviral vaccine inoculation was affected when a dose of 10^7^ pfu of adenovirus is pre-administered to these animals. However, the levels of pre-existing immunity generated after a dose of 10^7^ pfu of adenovirus, still allowed the induction of approximately 50% of specific lysis mediated by CMV-specific T-cells in immunized animals after i.n. pre-immunization. The specific lysis from both i.n. and i.m. groups was normalized with the positive control in which animals were vaccinated with a single dose of Ad5GFP in absence of adenovirus pre-existing immunity. The negative control (non-immunized mice) was used to verify that ^low^CFSE and ^high^CFSE+^CMV^CD8-peptide labeled cells were transferred into mice at comparable numbers.

In addition, prior application (i.n. or i.m.) of Ad5GFP at a dose of 10^3^ or 10^5^ pfu did not disturb the immunogenicity of the adenoviral vaccine. 92% and 82.5% of vaccinated animals pre-immunized i.n. with either 10^3^ or 10^5^ pfu of adenovirus, respectively, showed cytotopathic effects and 74% and 76% of those pre-immunized i.m. with either 10^3^ or 10^5^ pfu of adenovirus, respectively, showed cytopathic effects (Fig 1B). These results show that pre-established anti-Ad5GFP immunity mediates a dose-dependent interference in the immunogenicity of the adenoviral vaccine.

Prime-boost vaccination protocol overcomes adenovirus-specific pre-existing immunity and induces effective immune responses against the adenovirus vector-encoded transgene product.

**Fig 2:**
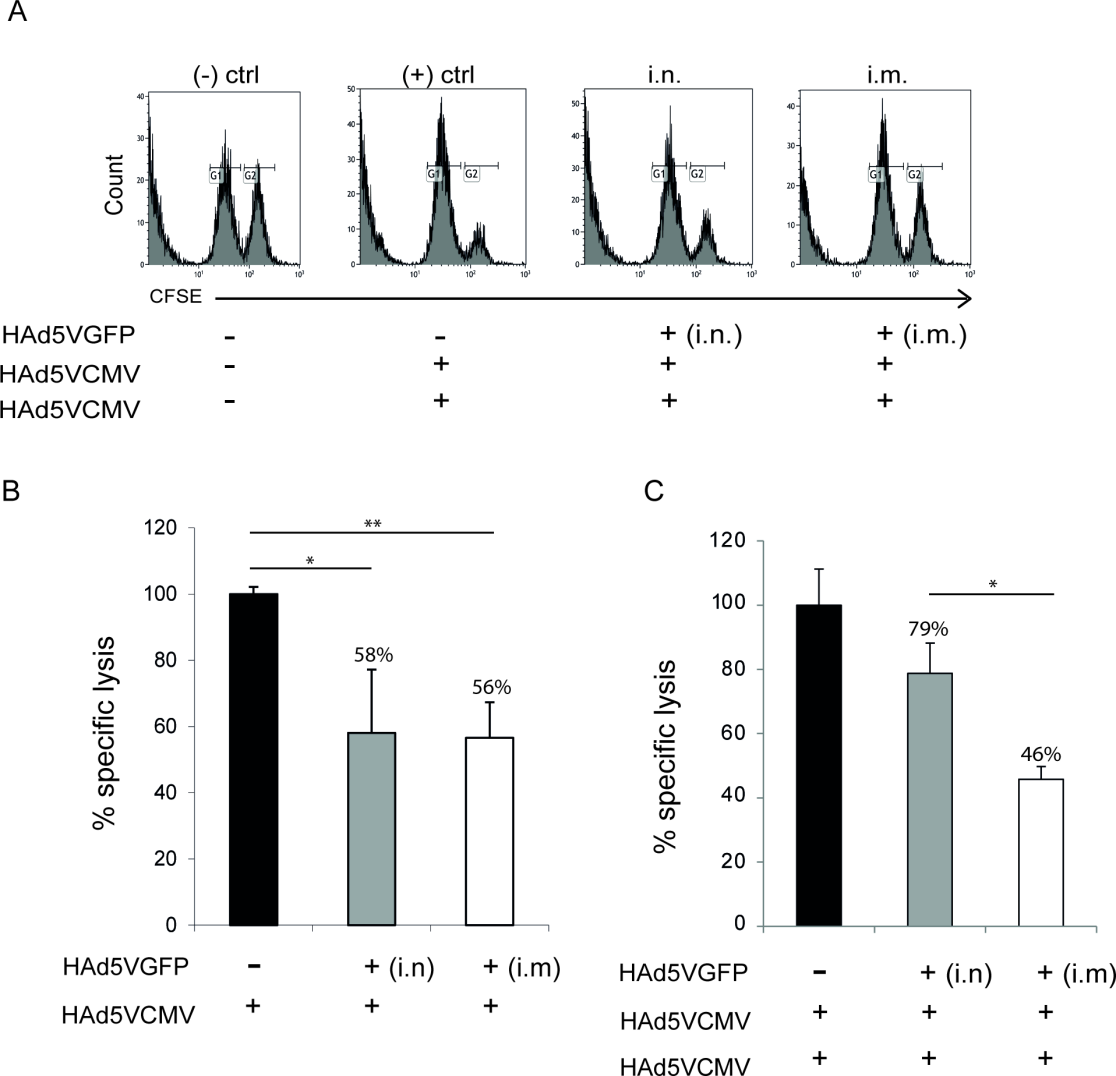
*In vivo* cytotoxicity levels against the murine CMV CD8 T-cell epitope YPHFMPTNL detected in animals with pre-existing HAd5V immunity or control animals after prime-boost immunization with HAd5V-based vaccine vector, HAd5VCMV. (A) Histograms show data from a single mouse and represent ^low^CFSE and ^high^CFSE+^CMV^CD8-peptide labeled cells detected in spleens of HAd5VCMV-immunized BALB/c mice or control animals. (B) Percentage of specific lysis induced in vaccinated animals after a single immunization (prime) or (C) after completion of a prime/boost protocol. Pre-immunity was induced with 1×10^7^ pfu/animal of Ad5GFP by the indicated route and vaccination was performed using one (prime) or two (prime/boost) doses (6 weeks apart) with 1×10^9^ pfu/animal of HAd5VCMV administered subcutaneously. The graphs in panel (B) and (C) show one of three independent experiments with at least 4 mice per group. Bars represent mean values +/- SD. (+) ctrl–positive control; (-) ctrl–negative control; (-) mice non-immunized with adenovirus; i.n.–intranasal immunization route; i.m.–intramuscular immunization route.

*In vivo* cytotoxicity assays were performed to analyze the effect of i.n. or i.m. induced pre-existing NAbs on a second immunization with HAd5VCMV (homologous prime/boost protocol, with a six-week interval between doses). After 20 hours, the presence of ^low^CFSE and ^high^CFSE+^CMV^CD8-peptide labeled cells in the spleen was analyzed by flow cytometry using the gates shown in Fig 2A. In Fig 2B we repeated the single-dose vaccination of the mice pre-immunized with Ad5GFP as a control for the group of animals vaccinated with the prime-boost immunization protocol. Interestingly, the animals with pre-existing immunity mounted different immune responses depending on the route of administration used to induce the pre-existing immunity after a prime-boost immunization protocol (Fig 2C). Animals that mounted an immune response against HAd5V by a natural route (i.n.) and were further vaccinated with two doses of HAd5VCMV induced significantly higher levels of antigen-specific CD8^+^ T cell lysis (78.8%) than animals that mounted an anti-adenoviral immune response by an unnatural route (45.8%). Furthermore, after administration of a booster vaccine dose, we did not find a significant statistical difference between the group of animals with i.n.-induced pre-existing immunity and animals in the control group, suggesting that a prime/boost protocol can be used as an efficient vaccination protocol even in presence of pre-existing Ad immunity generated by natural infections. The negative control of the experiment (non-immunized mice) was used in every antigen-specific cytotoxicity assay, specifically to monitor that comparable numbers ^low^CFSE and ^high^CFSE+^CMV^CD8-peptide labeled cells were transferred.

**Fig 3:**
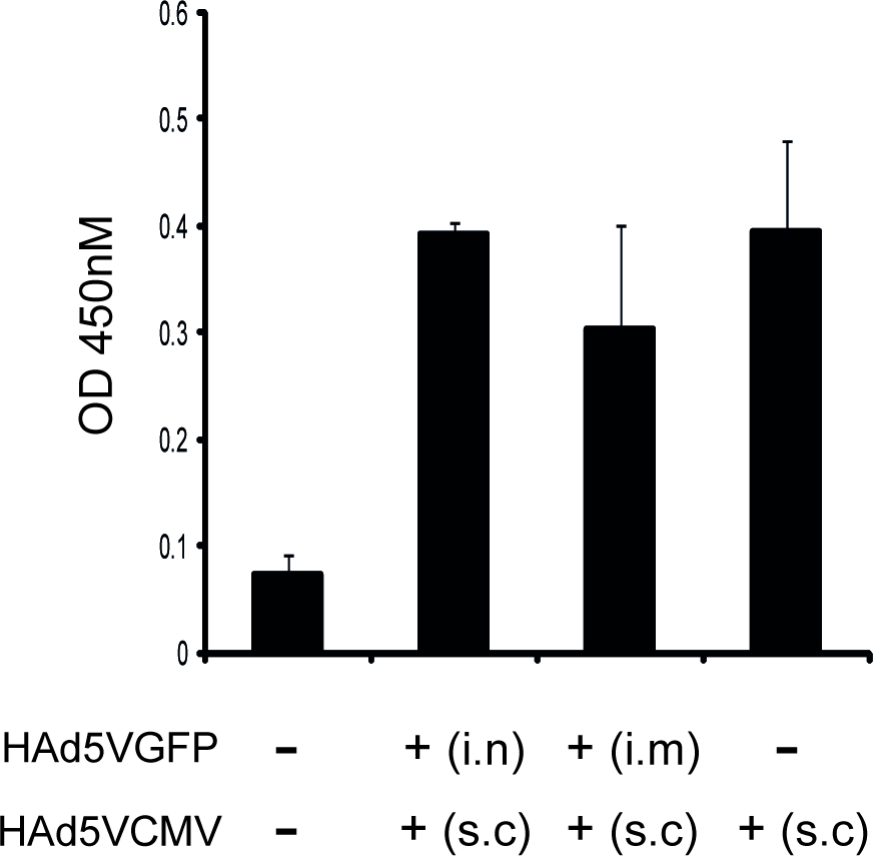
Humoral immune response to HBc protein following HAd5VCMV vaccination of HAdV pre-immunized animals or control animals. Pre-existing adenovirus-specific NAbs were induced by immunizing mice with HAd5VGFP (1x10^7^ PFU/animal) by either i.n. or i.m. route as indicated. Four weeks later, mice were subcutaneously immunized with HAd5VCMV (3x10^9^ PFU/animal). Control groups were injected with PBS (-) or HAd5VCMV when indicated. Serum samples were collected five days after the last immunization and analyzed by ELISA. The graph shows one of three independent experiments with at least 3 mice per group. Bars represent mean values +/- SD. (-) mice non-immunized with adenovirus; i.n.–intranasal immunization route; i.m.–intramuscular immunization route; s.c.–subcutaneous immunization route. *p = 0.0329; **p = 0.0077; ***p<0.0001.

Serum samples from HAd5V-pre-immunized mice vaccinated with a prime/boost protocol were also collected and analyzed by ELISA to determine any influence of the pre-existing immunity on the levels of humoral responses induced against HBc, the carrier protein used to generate the CMV vaccine. The analysis of the serum samples for non-immunized mice (-/-) revealed no significant anti-HBc antibody levels (Fig 3). In contrast, serum samples from animals immunized with HAd5VCMV after i.n. (i.n./s.c.) or i.m. (i.m./s.c.) induction of pre-existing anti-adenoviral immunity and sera from immunized animals without pre-existing immunity (-/s.c.) showed higher levels of anti-HBc antibodies than the sera of non-immunized mice (-/-) (Fig 3). These results are consistent with what was observed for T-cell immunity, and suggest that pre-existing immunity against adenovirus did not significantly affect the induction of humoral immune responses against the adenovirus vector-mediated transgene product.

### Induction of Ad5NAbs in mice through the natural and experimental route

**Fig 4:**
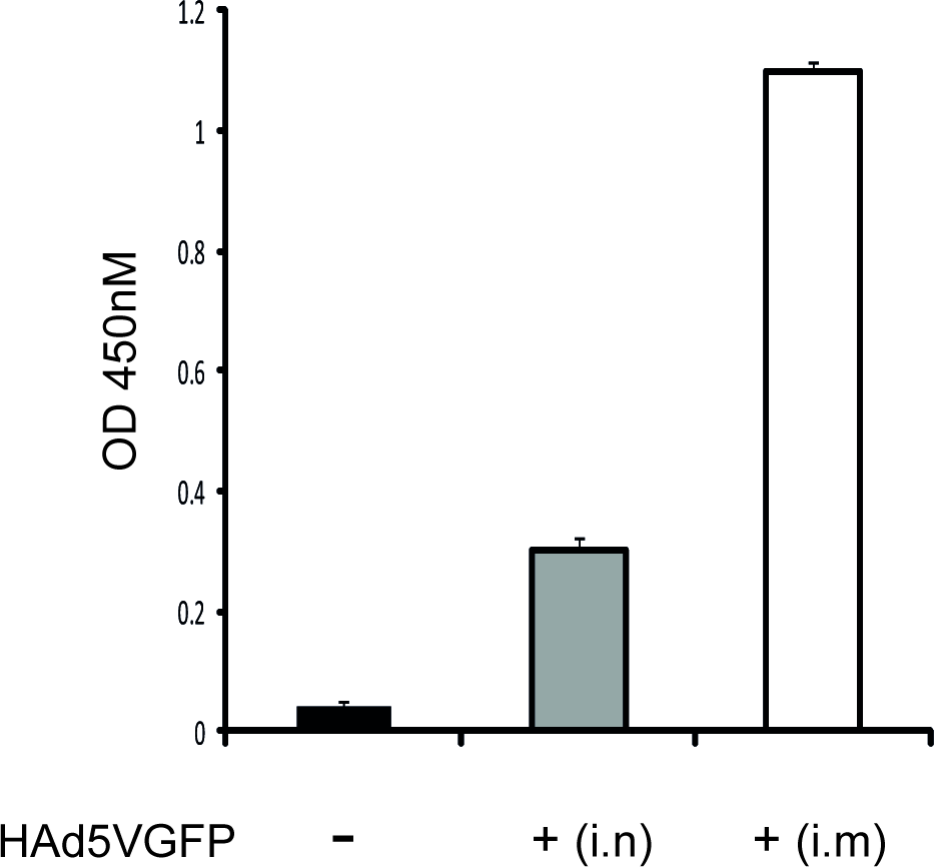
Humoral immune responses to HAd5VGFP detected in serum samples of mice immunized with HAd5VGFP by i.n. or i.m. inoculation route. Mice were immunized with HAd5VGFP (10^7^ pfu/animal) by i.n. or i.m. route. One week later, sera were collected and the anti-HAd5VGFP antibodies were measured by ELISA. The control group was injected with PBS (-). The graph shows one of two independent experiments with at least 4 mice per group. Bars represent mean values +/- SD. (-) mice non-immunized with adenovirus; i.n.–intranasal immunization route; i.m.–intramuscular immunization route; s.c.–subcutaneous immunization route.

To analyze the difference between adenovirus specific pre-existing immunity induced by either i.n. or i.m. inoculation route that might have accounted for the differences observed in the immune responses after prime-boost vaccination with adenovirus vectors, we analyzed the humoral immune responses against the adenovirus vector after i.n. or i.m. infection. Fig 4 shows that the anti-Ad5GFP antibody levels were lower in mice immunized i.n. with Ad5GFP (10^7^ pfu) than in mice immunized by i.m. route.

We also analyzed by neutralization assay the Ad5Nabs induced by either i.n. or i.m. inoculation route. As shown in Table 1, titers of Ad5NAbs below 200 NU were obtained when 10^7^ pfu of HAd5VGFP per animal were inoculated by the i.n. route. The same dose of virus inoculated by the i.m. route triggered a NAb titer of over 1000 NU. Even a dose of 10^5^ pfu/animal triggered levels of NAbs at medium-to-high levels (200–1.000 NU) when given by the parenteral route. These results suggest that i.m. inoculation induces higher levels of NAbs that diminish the immunization efficacy of adenovirus-based vaccine vectors, while the lower NAb levels induced by i.n. inoculation is overcome by prime-boost immunization protocols.

**Table 1:** HAd5V dose-dependent neutralizing antibody titers detected in BALB/c mice.

Inoculum PFU/animal	Neutralization titer (NU)
10^3^ i.n.	<20
10^5^ i.n.	<20
10^7^ i.n.	20–200
10^3^ i.m.	20–200
10^5^ i.m.	200–1000
10^7^ i.m.	>1000
neg (mouse)	<20
pos (human)	200–1000
neg (human)	<20

Serum samples were collected from animals previously inoculated by i.n. or i.m. route with the indicated dose of HAd5VGFP vector. The Ad5NAb titers were compared with human and mouse serum controls. i.n.–intranasal immunization route; i.m.–intramuscular immunization route; neg–negative HAd5V serum sample; pos–positive HAd5V serum sample; NU–neutralizing unit.

**Fig 5:**
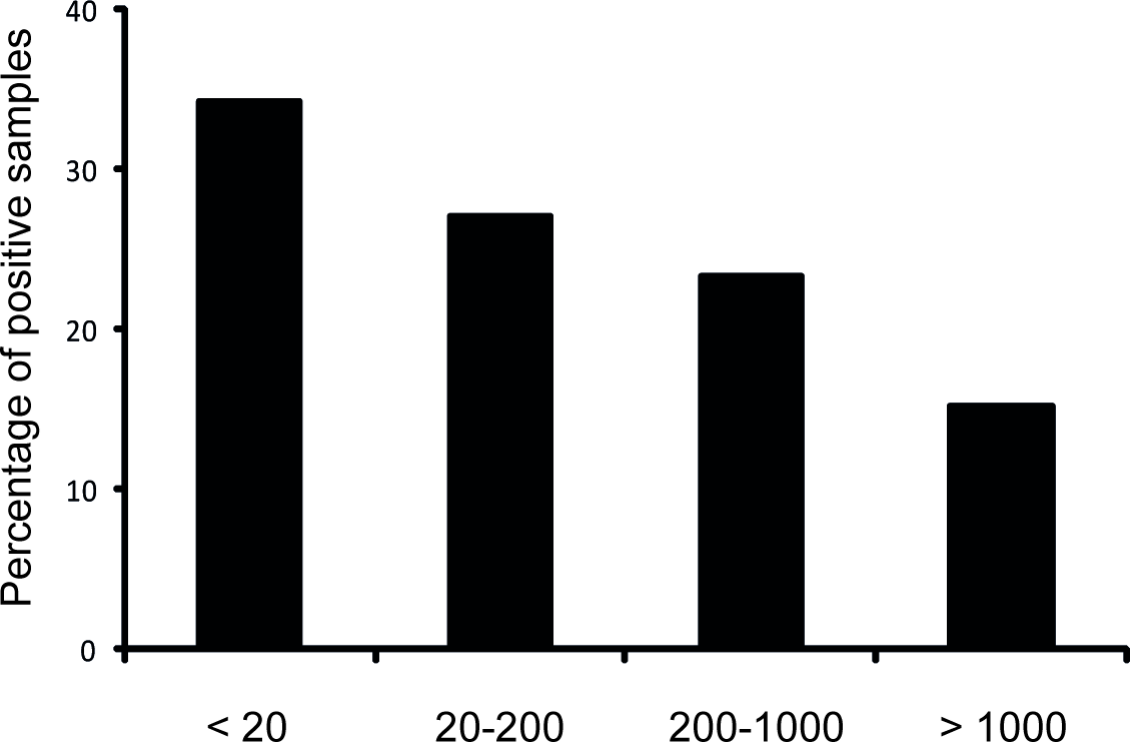
Neutralizing antibody titers detected in serum samples of inhabitants of two different ethnic regions of Brazil. Percentage of anti-HAd5V positive human serum samples from Visconde do Rio Branco (Southeastern State of Minas Gerais, 103 samples) and Belem (Northern State of Pará, 107 samples) inhabitants. The results are represented as the 4 most significant ranges of neutralizing antibody titers.

To indirectly correlate the Ad5NAb-levels found in mice with human Ad5NAb-levels, we analyzed the prevalence and titers of Ad5NAbs in sera of 210 inhabitants from two different regions of Brazil -North and Southeast. Results show that independently of the origin of the sera analyzed, the levels of anti-HAd5V antibodies detected were very similar in both regions (data not shown). In summary, Fig 5 shows that 34.3% of the samples of Brazilian inhabitants tested had no quantifiable anti-HAd5V NAbs (titers <20 NU), 27.1% had low levels (titers between 20 and 200 NU), 23.3% displayed medium to high levels (titers between 200 and 1.000); and 15.2% had high levels of antibodies (titers >1.000 NU). Considering that animals injected i.n. with adenovirus vectors generate pre-existing Ad5NAb-levels of 20–200 NU, which did not impair the prime-boost immunization, we suggest that humans that display HAd5V neutralizing activity at lower levels (34.3%) and at the same levels of 20–200 NU (27.1%) could be efficiently vaccinated with adenovirus vectors.

## Discussion

Of interest, our data on routes and doses required for induction of pre-existing immunity suggest that the i.n. and i.m. routes behave very differently, since equivalent doses of Ad5GFP induced drastically different levels of NAbs after using one or the other route of administration. Previous studies have also demonstrated that cellular[29] and humoral immune responses[30] vary according to adenovirus dose and administration route. Since the natural route of infection with HAd5V is the respiratory route, and most previous studies induced pre-existing immunity by inoculation of HAd5V vectors by a parenteral route, some previous conclusions may have been extracted from an experimental setting that does not represent the actual human situation. In contrast to our findings Pandey et al. showed that pre-existing NAbs induzed in mice by natural adenovirus infection (i.n.) can be overcome even with a single dose of adenovirus vector based immunization. This difference might be explained by the higher doses of adenovirus used for immunizations in the study by Pandey and colleagues. Indeed, those authors showed that even in presence of vector immunity a humoral immune response to the target antigen was induced by increasing the vaccine dose.[30]

We are aware that immune responses elicited in the animals may not be identical to those induced in humans, i.e. the virus used in our experiments is a human virus for which the mouse is an unnatural host. However, the positive results obtained after using a prime-boost immunization protocol of the HAd5VCMV vaccine in animals with a human-like i.n.-induced pre-existing immunity, suggests that most conclusions obtained in experimental models in which pre-existing immunity was induced by an unnatural route may not represent the real situation observed in humans, the ultimate goal of our vaccination efforts. Differences found in the interference of pre-existing immunity in the induction of immune responses could be explained also by differences in viral doses used to induce NAbs. We have shown here that differences in the adenovirus doses correlate with the immune responses generated by adenovirus vector vaccines. The lower doses of Ad5GFP (10^3^ and 10^5^ PFU/animal) used to induce adenovirus-specific pre-existing antibodies generated significantly higher levels of antigen-specific CD8^+^ T cell lysis than animals pre-immunized with the highest dose of Ad5GFP (10^7^ PFU/animal) independently of the route of infection (i.n. or i.m.).

HAd5V has been extensively studied as a gene therapy vector to cure human diseases. The great potential of using HAd5V for gene transfer is compromised by the concern of vector neutralization due to the high levels of Ad5NAbs present in humans worldwide. Here, we have demonstrated that a prime-boost protocol of vaccination can efficiently overcome the anti-HAd5V-specific pre-existing immunity.

Regarding the applicability of our results to assist in decisions on HAd5V-based vaccine usage, it is currently believed[19,31] that individuals with antibody titers below 200 NU display a low level of adenoviral particle neutralization and could be treated with adenoviral vectors. This considered, 61.4% of the Brazilian population could possibly be vaccinated with a recombinant HAd5V vector regardless of adenovirus-specific pre-existing immunity. We assume that up to 60% of the Brazilian population with HAd5V/NAb titers of > 200 NU (38.6%) could be immunized effectively with HAd5V vectors as shown in the Merck STEP study [19]. However, additional analyses of efficiency and safety in this population are needed. Human trials are currently being performed with HAd5V vectors by different research groups worldwide in which these aspects will likely be considered.[32,18]

It is worth noting that our conclusions are extracted from analyses performed with sera of inhabitants of Brazil, irrespective of any selection criteria such as age or health status. Thus, it is feasible to believe that we are considering the worst possible setting, and that in case of a more precise selection of a target population, i.e. children between 6 months to 2 years of age, for which the levels of anti-HAd5V have previously been described to be much lower than for adults,[31,33] a recombinant adenoviral vaccine should be expected to be even more efficient. Most recent studies have confirmed the relatively low levels of human adenovirus NAbs in 7–12 months old children, an age range that may provide a vaccination window. This setting is the most adequate for diseases like malaria, leishmaniosis, diarrhoea and other infectious diseases, for which the corresponding vaccines should be included in the children’s national immunization schedules. It is important to note that adenovirus-specific NAbs generated by adenovirus vectors could eventually reduce the efficacy of future vaccinations using the same vector. Therefore, the use rare human serotype adenovirus vectors or modified adenovirus vectors may be considered.

Currently, several independent groups have demonstrated the efficacy of HAd5V-vector vaccines in clinical trials. Morse and colleagues[18] used adenovirus subtype 5 vectors (E1- and E2b- regions deleted) in patients with metastatic colorectal cancer to efficiently induce tumor antigen-specific cell-mediated immune responses despite of the presence of significant levels of either pre-existing HAd5V-specific neutralizing antibodies or vector induced HAd5V immunity. Moreover, in this study revealed that patients treated with HAd5V had no serious side effects after three administrations of 1x10^10^ particles of the HAd5V vaccine vector. Another clinical study using a DNA priming/HAd5V boosting regimen for inducing malaria-specific immune responses was able to induce protection against *P*. *falciparum* in 27% of volunteers. The protection was related to cell-mediated immunity and the protocol included the application of three doses of DNA plasmids and a single inoculation with a non-replicating recombinant human adenovirus vector. [32] The efficacy of HAd5V-based vectors was also demonstrated by Smaill and colleagues.[34] This study showed that HAd5V vectors encoding an immune dominant *Mycobacterium tuberculosis* antigen induced antigen-specific T cells even in patients with medium to high levels of pre-existing anti-HAd5V antibodies.[34]

In conclusion, we have shown that HAd5V-based prime/boost immunization regimes may overcome low levels of naturally-occurring vector-specific pre-existing immunity, as present in nearly 75% of the general Brazilian population. We believe that HAd5V-based immunization protocols may be even further optimized to avoid impairment of vaccination efficacy, for example by using less-prevalent adenovirus serotypes. Therefore, this study highlights the importance of continued assessment of HAd5V as a feasible vaccine vector in humans.
